# Real-world outcomes of lurasidone augmentation for treatment-resistant bipolar depression: a retrospective observational analysis

**DOI:** 10.3389/fpsyt.2026.1744056

**Published:** 2026-02-19

**Authors:** Giorgia Porceddu, Enrico Pessina, Carlo Ignazio Cattaneo, Vassilis Martiadis, Carolina Bianca, Giuseppe Maina, Gianluca Rosso

**Affiliations:** 1 Department of Neurosciences, San Luigi Gonzaga University Hospital, Turin, Italy; 2 Department of Mental Health, Community Mental Health Center, Local Health Unit of Cuneo 2, Cuneo, Italy; 3 Department of Mental Health, Local Health Unit of Biella, Biella, Italy; 4 Department of Mental Health, Local Health Unit of Napoli 1 Centro, Napoli, Italy; 5 Department of Neurosciences, University of Turin, Turin, Italy

**Keywords:** bipolar depression, bipolar disorder, hamilton depression rating scale, lurasidone, TRBD, treatment-resistance

## Abstract

**Introduction:**

Treatment-resistant bipolar depression (TRBD) is a major challenge in psychiatric practice, leading to marked impairment in functioning and quality of life, and increased healthcare utilization. Despite its clinical relevance, consensus on diagnostic criteria and evidence-based therapeutic strategies remains limited. Within the framework of personalized medicine, identifying effective and well-tolerated options for this heterogeneous population is important. This study evaluates the short-term effectiveness and tolerability of lurasidone as an adjunctive treatment in TRBD, with attention to its potential role in tailoring interventions to individual clinical profiles.

**Methods:**

This four-week, retrospective, multicentre observational study included patients with TRBD receiving lurasidone in augmentation to ongoing pharmacological treatment. Dosages were adjusted according to clinical judgement. Symptom severity was assessed with the 17-item Hamilton Depression Rating Scale (HAM-D), Young Mania Rating Scale (YMRS), and Hamilton Anxiety Rating Scale (HAM-A). Changes from baseline to endpoint were analyzed with repeated measures ANOVA; missing data were managed with the Last Observation Carried Forward (LOCF) method.

**Results:**

Sixty patients were enrolled. The mean final lurasidone dose was 51.2 mg/day. Significant improvements were observed across all scales, with consistent reductions in depressive and anxiety symptoms. Clinical response was achieved in 33.3% of participants, while remission occurred in 3.3%. Adverse events were reported by 68.3% of completers, all mild to moderate.

**Conclusions:**

Lurasidone appears to be an effective and generally well-tolerated adjunctive option for TRBD. However, remission rates remained low, underscoring the need for further research. In this perspective, lurasidone may contribute to more individualized treatment strategies for difficult-to-treat patients, although confirmatory studies are required to better define its role within precision psychiatry.

## 1 Introduction

Depressive episodes represent the primary clinical manifestation of bipolar disorder (BD), accounting for the majority of symptomatic periods and playing a central role in the condition’s overall morbidity and mortality (1, 2). These episodes are closely linked to marked impairments in psychosocial functioning, reduced quality of life, and a significantly heightened risk of suicide (3, 4), thereby presenting considerable challenges in clinical management.

The clinical heterogeneity of bipolar depressive episodes—often marked by mixed features, psychomotor agitation, and anxiety symptoms—further complicates both accurate diagnosis and effective treatment planning (5, 6). Misdiagnosis is common, particularly in the early stages of the illness, with bipolar depression often erroneously identified as unipolar major depressive disorder. Evidence suggests that high rates of individuals initially diagnosed with treatment-resistant unipolar depression may potentially be reclassified as having bipolar disorder within one year (7). Such diagnostic delays are clinically consequential, frequently resulting in inappropriate pharmacological interventions —most notably, antidepressant monotherapy— which may exacerbate mood instability and negatively impact long-term outcomes (8, 9).

Despite the considerable clinical burden associated with bipolar depression, there is still little attention to developing personalized pharmacological strategies tailored to the different clinical subtypes of the disorder. Treatment options remain limited, and to date only a small number of agents have received formal regulatory approval for this indication (10, 11). Among the currently recommended first-line treatments—namely quetiapine, lithium, lamotrigine, and the olanzapine–fluoxetine combination—therapeutic response rates are frequently suboptimal. Approximately 40% of patients treated with quetiapine over an eight-week period fail to demonstrate clinically meaningful improvement (12, 13). Response rates for other agents, including lithium, lamotrigine, and the olanzapine–fluoxetine combination, are often even lower (14–18). Thus, many real-world bipolar patients are actually treatment-resistant, but no standardized diagnostic criteria have yet been established for treatment-resistant bipolar depression (TRBD). Various definitions have been suggested, typically grounded in a history of inadequate response to conventional pharmacotherapies (19–22). However, the applicability of these criteria is constrained by the exclusion of certain authorized or by the absence of regulatory approval for several listed medications in various countries.

Lurasidone is an atypical antipsychotic characterized by high-affinity antagonism at 5-HT_2_A, 5-HT_7_, and D_2_ receptors, partial agonism at 5-HT_1_A receptors, and minimal affinity for H_1_ and M_1_ receptors (23). This receptor profile could potentially contribute not only to antipsychotic effects but also to mood-stabilizing and antidepressant properties. However, clinical evidence supporting such effects in bipolar depression remains limited and is largely restricted to patients with bipolar disorder type I (24–26). In Europe, lurasidone is approved exclusively for the treatment of schizophrenia. In the United States, it is approved for both schizophrenia and depressive episodes associated with bipolar I disorder. Data specifically addressing its use in treatment-resistant bipolar depression (TRBD) remain scarce. The present study aimed to evaluate the real-world effectiveness and tolerability of lurasidone in patients with TRBD.

## 2 Materials and methods

### 2.1 Study design and patients

A retrospective, multicentre, observational study was conducted to evaluate the short-term effectiveness and tolerability of lurasidone when used as an adjunctive therapy in patients diagnosed with treatment-resistant bipolar depression (TRBD).

Medical records were reviewed for both inpatients and outpatients diagnosed with bipolar disorder, according to Diagnostic and Statistical Manual of Mental Disorders, Fifth Edition, Text Revision (27) criteria, who were consecutively admitted or referred to one of the participating psychiatric centres between January and May 2025. These centres included: the Psychiatric Unit of San Luigi Gonzaga University Hospital of Orbassano (University of Turin, Italy); the Mental Health Departments of Alba and Bra (Cuneo, Italy); the Department of Mental Health of Biella (Biella, Italy); and the Department of Mental Health of Napoli 1 (Naples, Italy).

Patients included met the follow criteria: (a) age ≥18 years; (b) a confirmed primary diagnosis of bipolar disorder based on DSM-5-TR criteria; (c) presence of a current major depressive episode, including episodes with anxious, mixed, or psychotic features; (d) presence of treatment resistance, defined operationally in accordance with Murphy and colleagues (28). This definition was selected for several reasons: it represents one of the earliest and most widely cited operational attempts to define TRBD and has been adopted in several subsequent clinical and naturalistic studies, allowing comparability with existing literature (29, 30); it also provides a pragmatic and clinically applicable framework suitable for real-world settings, such as the present naturalistic study. Treatment resistance was defined as an insufficient response to at least two mood stabilizer classes (including atypical antipsychotics) and two antidepressant classes, with each pharmacological trial deemed adequate in terms of both dosage and duration. Current guidelines for bipolar depression emphasize mood stabilizers and atypical antipsychotics and recommend caution with antidepressant use; however, antidepressants are frequently prescribed in routine clinical practice, particularly in patients with complex or refractory presentations. Inclusion of antidepressant non-response in this definition therefore reflects real-world treatment trajectories rather than an endorsement of antidepressant-centred strategies, and allows capture of a clinically meaningful population with documented insufficient response to multiple pharmacological interventions (29, 30); (e) ongoing treatment with at least one mood stabiliser—lithium, valproate, or lamotrigine—maintained within therapeutic plasma levels; and (f) initiation of lurasidone as an adjunctive agent to the existing pharmacotherapy. The starting dose of lurasidone, along with any subsequent titration, was determined at the discretion of the treating psychiatrist, based on individual clinical presentation and judgement.

The study protocol received approval from the local Ethics Committee. All participants provided written informed consent permitting the anonymous use of their clinical data for research purposes.

### 2.2 Assessment and procedures

Socio-demographic, clinical, and safety-related information was obtained from patients’ medical records. Follow-up assessments were conducted in line with routine clinical practice. The severity of psychiatric symptoms was evaluated in an unblinded manner using the 17-item Hamilton Depression Rating Scale (HAM-D), the Hamilton Anxiety Rating Scale (HAM-A), and the Young Mania Rating Scale (YMRS).

All psychiatric diagnoses and clinical assessments were performed by consultant psychiatrists with substantial clinical expertise.

The primary measure of treatment effectiveness was the mean change in HAM-D scores from baseline to the end of the four-week observation period. In addition, a qualitative analysis was conducted, defining rates of treatment response as a reduction of ≥50% in HAM-D scores, and remission as achieving a HAM-D score below 7.

### 2.3 Statistical analysis

Sociodemographic and clinical variables were described using means and standard deviations for continuous measures, and percentages for categorical variables. A *post hoc* power analysis, based on a sample size of 60 participants and a significance level of 0.05, indicated a statistical power exceeding 95% for detecting changes in mean HAM-D and HAM-A scores (Cohen’s *d* = 1.92 for HAM-D reduction; *d* = 1.14 for HAM-A reduction).

Since baseline data followed a normal distribution (Kolmogorov–Smirnov test: D = 0.094; p = 0.20; Shapiro-Wilk test: D = 0.975; p = 0.25), parametric statistical methods were applied. Changes in clinical rating scale scores across the four-week observation period were examined using repeated measures analysis of variance (rm-ANOVA). Three distinct rm-ANOVA models were constructed, with the HAM-D, HAM-A, and YMRS scores as dependent variables, to assess potential interaction effects over time. The assumption of sphericity for the covariance matrix was evaluated using Mauchly’s test; where this assumption was violated, the Greenhouse–Geisser epsilon (ϵ) correction was applied to adjust the degrees of freedom accordingly. Missing data were addressed using the Last Observation Carried Forward (LOCF) method.

Participants were subsequently categorized as responders or non-responders, based on a ≥50% reduction in HAM-D scores. Comparisons between the two groups were performed using the t-test for continuous variables and the χ² test for categorical variables.

Participants were further stratified into two subgroups according to the presence of moderate-to-severe anxiety, defined by a HAM-A score ≥25. The two subgroups were then compared with respect to response versus non-response to lurasidone using the χ² test.

Statistical significance was set at a p-value of <0.05. All analyses were carried out using IBM SPSS Statistics, version 28.0.1.

## 3 Results

A total of sixty individuals met the inclusion criteria and were subsequently enrolled in the study cohort. Of these, 29 individuals (48.3%) were female. The cohort had a mean age of 44.9 ± 15.0 years. Regarding diagnostic classification, 29 participants (48.3%) met criteria for bipolar disorder type I, while the remaining 31 (51.7%) were diagnosed with bipolar disorder type II. The mean age at illness onset of was 26.5 ± 8.7 years. Comorbid psychiatric conditions were identified in 27 patients, accounting for 45% of the cohort.

Nearly all participants (n: 57; 95.0%) were receiving pharmacological treatment with mood stabilizing agents at baseline. Of these, approximately 10% were prescribed a combination regimen involving two mood stabilizers. Additionally, 40 patients (66.7%) were concurrently treated with at least one antidepressant, and 17 individuals (28.3%) were receiving antipsychotic medications. Baseline clinical assessment revealed a mean score of 25.9 ± 4.3 on the Hamilton Depression Rating Scale (HAM-D), indicative of severe depressive symptoms, and a mean score of 24.5 ± 7.1 on the Hamilton Anxiety Rating Scale (HAM-A), consistent with moderate anxiety severity. A comprehensive overview of the socio-demographic and clinical characteristics of the sample at baseline (**Table 1**).

**Table 1:** Baseline demographic and clinical characteristics of the sample.

Parameters	N: 60
**Age**, years (mean ± SD)	44.9 ± 15.0
**Sex, *n* (%)**	
Male	31 (51.7)
Female	29 (48.3)
**Marital status, *n* (%):**	
Single	27 (45.0)
Married	25 (41.7)
Divorced	7 (11.7)
Widowed	7 (1.7)
**Educational level,** years (mean ± SD)	12.9 ± 3.1
**Occupational status, n (%)**	
Student	6 (10.0)
Unemployed	28 (46.7)
Employed	14 (23.3)
Housekeeper	7 (11.7)
Retired	5 (8.3)
**Age at onset of bipolar disorder,** years (mean ± SD)	26.5 ± 8.7
**Bipolar disorder, type, *n* (%)**	
BD I	29 (48.3)
BD II	31 (51.7)
**Lifetime suicide attempts**, n (%)	27 (45.0)
**Family history of psychiatric disorders**, n (%)	31 (51.7)
**Lifetime Psychiatric Comorbidities**, *n* (%)	27 (45.0)
**Type of psychiatric comorbidities, *n* (%)**	
Obsessive-compulsive disorder	5 (8.3)
Anxiety disorders	19 (31.6)
Substance use disorders	3 (5.1)
**Current mood stabilizer**, *n* (%)	57 (95.0)
**Class of mood stabilizer, *n* (%)**	
Lithium	30 (50.0)
Valproate	14 (23.3)
Lamotrigine	3 (5.0)
Combination of two mood stabilizers	10 (16.7)
**Current antidepressant**, *n* (%)	40 (66.7)
**Current antipsychotics**, *n* (%)	17 (28.3)
**HAM-D scores**, (mean ± SD)	25.9 ± 4.3
**YMRS scores**, (mean ± SD)	4.7 ± 3.3
**HAM-A scores**, (mean ± SD)	24.5 ± 7.1
**BPRS scores**, (mean ± SD)	30.3 ± 4.1

SD, standard deviation; BD I, Bipolar Disorder type I; BD I, Bipolar Disorder type II; HAM-D, Hamilton Depression Rating Scales; YMRS, Young Mania Rating Scale; HAM-A, Hamilton Anxiety Rating Scale; BPRS, Brief Psychiatric Rating Scale.

The mean daily dose of lurasidone prescribed at baseline was 32.9 mg/day. Over the 4-week observation period, the mean dose increased to 46.7 mg/day, reaching a mean final dose of 51.2 mg/day. The overall dose range throughout the study was 18–111 mg/day.

**Figure 1:**
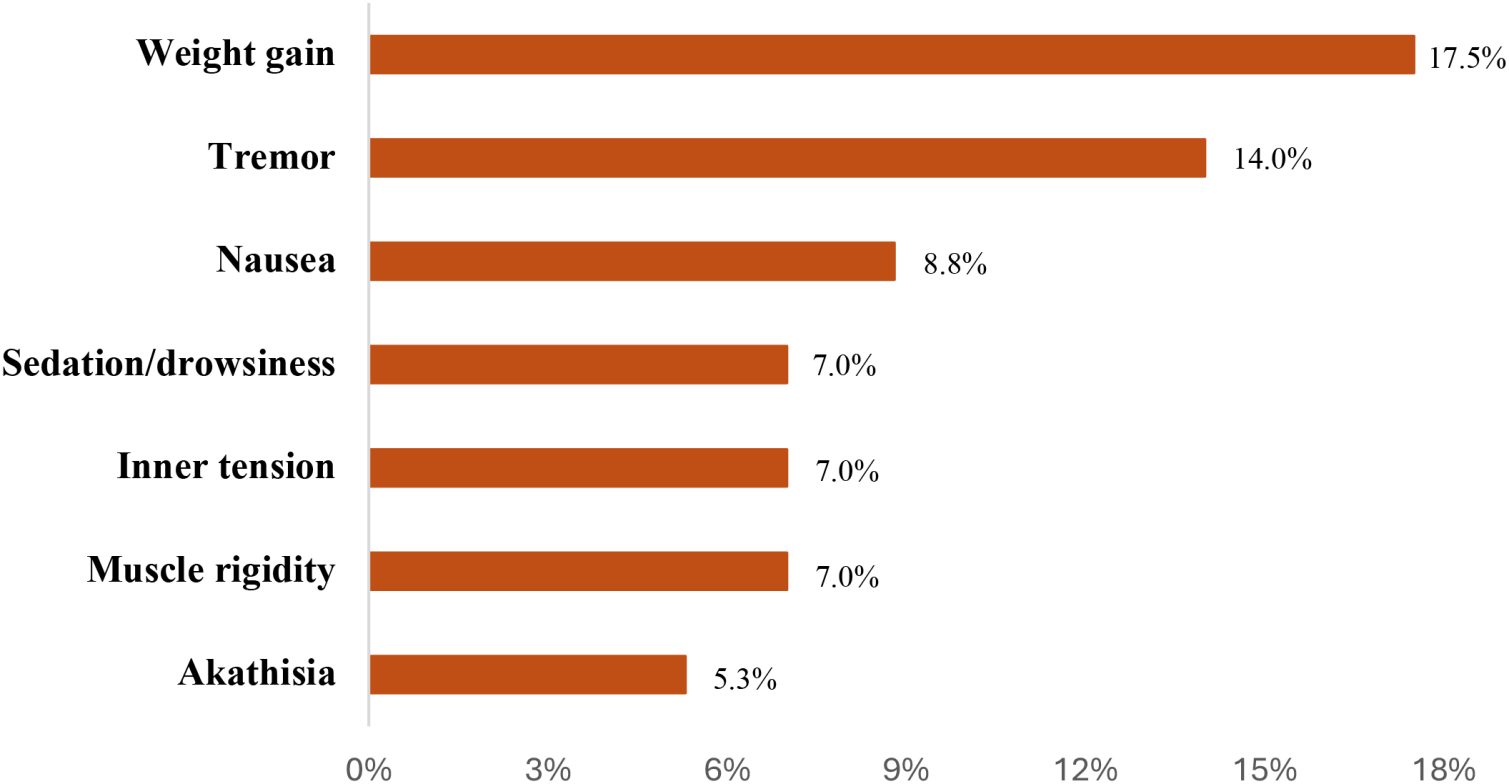
Most common adverse events. Adverse events <5%: acute dystonia, urinary difficulties, emotional blunting, leg oedema, and nocturnal hyperhidrosis.

Among the participants, 41 (68.3%) experienced at least one treatment-emergent adverse event (**Figure 1**).

**Figure 2:**
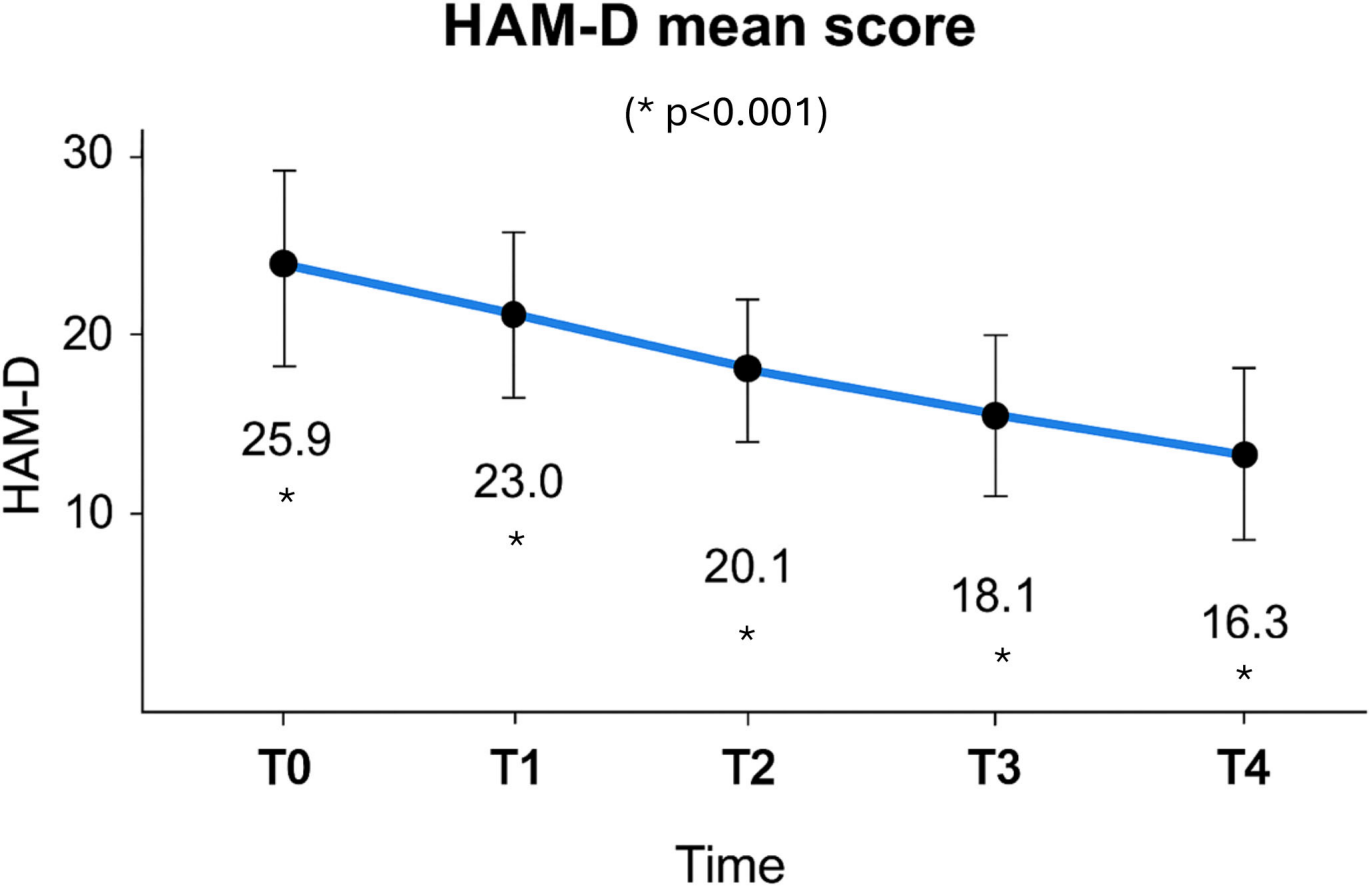
Mean reduction of Hamilton Depression Rating Scale (HAM-D) scores during follow-up. The following abbreviations are used in this manuscript: Bipolar Disorder (BD); Treatment-resistant Bipolar Depression (TRBD); Hamilton Depression Rating Scale (HAM-D); Young Mania Rating Scale (YMRS); Hamilton Anxiety Rating Scale (HAM-A); repeated measures analysis of variance (rm-ANOVA); Last Observation Carried Forward (LOCF); Diagnostic and Statistical Manual of Mental Disorders, Fifth Edition, Text Revision (DSM-5-TR); SD: standard deviation.

A reduction in the mean HAM-D score was observed from baseline (T0) to week 4 (T4), with progressive improvement noted at each subsequent assessment. Specifically, mean scores decreased from 25.9 ± 4.3 at T0 to 23.0 ± 5.4 at T1 (ΔM = 2.847, S = 0.401, p <0.001), 20.01 ± 5.7 at T2 (ΔM = 5.915, SE = 0.586, p < 0.001), 18.1 ± 6.2 at T3 (ΔM = 7.932, SE = 0.587, p <0.001), and finally 16.3 ± 6.9 at T4 (ΔM = 9.576, SE = 0.656, p <0.001) (**Figure 2**).

Reductions from baseline were observed at all subsequent time points for the HAM-A score. Mean values decreased from 24.5 ± 7.1 at baseline (T0) to 22.4 ± 7.7 at T1 (ΔM = 2.136, SE = 0.352, p < 0.001), 20.6 ± 8.7 at T2 (ΔM = 4.068, SE = 0.536, p < 0.001), 19.3 ± 9.2 at T3 (ΔM = 5.441, SE = 0.668, p < 0.001), and 18.0 ± 10.0 at T4 (ΔM = 6.492, SE = 0.752, p < 0.001).

Similarly, the YMRS scores showed a decrease over time, from a baseline mean of 4.7 ± 3.3 to 3.6 ± 3.2 at T1 (ΔM = 1.088, SE = 0.226, p < 0.001), 3.0 ± 3.2 at T2 (ΔM = 1.737, SE = 0.393, p < 0.001), 2.1 ± 2.6 at T3 (ΔM = 2.702, SE = 0.391, p < 0.001), and 1.9 ± 2.7 at T4 (ΔM = 2.860, SE = 0.437, p < 0.001).

All findings were confirmed by the Last Observation Carried Forward (LOCF) analysis, which resulted in consistent findings across all time points.

By the end of the observation period, 20 out of 60 patients (33.3%) met criteria for clinical response, defined as a ≥50% reduction in HAM-D scores. Remission, defined as a HAM-D score <7, was achieved by 2 patients (3.3%).

When comparing responders (n: 20; 33.3%) and non-responders (n: 40; 66.7%), no statistically significant differences were found in key sociodemographic variables, including age and years of education, nor in core clinical characteristics such as bipolar disorder subtype and age at illness onset. Baseline scores on the HAM-D, HAM-A, and YMRS did not significantly differ between groups (p: 0.418, p: 0.253, and p: 0.109, respectively), indicating comparable levels of depressive, anxious, manic, and psychotic symptoms at study entry.

When comparing patients with moderate-to-severe anxiety (n: 34; 56.7%) and those with mild anxiety (n: 26; 43.3%), no statistically significant differences emerged in response rates to lurasidone treatment (p: 0.461). The same applies in the response rates when comparing low (< 74 mg/d) or high (≥ 74 mg/d) doses of lurasidone according to the presence/absence of moderate-to-severe anxiety (respectively, p: 0.154 and p: 0.871).

Regarding lurasidone augmentation, responders received a significantly higher initial dose compared to non-responders (39.5 ± 19.5 mg vs 29.6 ± 15.6 mg; p: 0.037). However, no significant between-group difference was found in the lurasidone dose at the end of the observational period (p: 0.219).

Regarding treatment-related characteristics, a significant association was found between the use of dual mood stabilizers and clinical response (p: 0.007). Patients receiving more than one mood-stabilizing agent were more likely to achieve a ≥50% reduction in HAM-D scores (70%) compared to those treated with a single mood stabilizer (26%). Similarly, antipsychotic use was significantly associated with clinical response (p: 0.008), with a higher proportion of patients receiving antipsychotics showing a clinical response (58.8%) relative to those who did not (23.2%). A significant association also emerged between antidepressant use and clinical response (p: 0.012); unexpectedly, patients who did not receive antidepressants were more likely to respond to treatment (55%) than those who did (22.5%).

## 4 Discussion

Treatment-resistant bipolar depression (TRBD) represents a significant and often under-recognised public health issue. While bipolar disorder (BD) is a leading cause of global disability, a subset of patients who do not adequately respond to standard treatments frequently experience a particularly chronic and debilitating course, with significant impacts on quality of life, psychosocial functioning, and long-term outcomes (31–33). Individuals with TRBD often suffer from prolonged depressive episodes, elevated suicide risk, diminished occupational and social engagement, and poor treatment adherence—factors that contribute to increased hospitalization, unemployment, and a range of psychiatric and physical comorbidities (34).

Despite the urgent demand for effective therapeutic strategies, the clinical management of TRBD remains challenging and inconsistently standardized. This is largely due to the absence of universally accepted diagnostic criteria and clearly established treatment guidelines (35). Such lack of consensus not only complicates clinical decision-making but also hinders research efforts, limiting accurate assessment of the condition’s prevalence and obstructing the advancement of targeted interventions. Although estimates vary widely depending on the criteria applied, existing data indicate that approximately one-quarter of individuals with BD may experience treatment-resistant depressive episodes (17, 31).

In this context, lurasidone has emerged as a noteworthy pharmacological option. Classified as a second-generation antipsychotic, it has demonstrated efficacy and safety in the treatment of bipolar depression (25, 26, 36) and is included as a first-line option in international clinical guidelines (10, 37). However, despite this evidence, its use for bipolar depression remains off-label in European countries due to regulatory limitations.

Based on the outcomes of our study, lurasidone demonstrated an effectiveness in reducing anxiety and depressive symptoms over a four-week treatment period. The progressive reduction in mean scores across all assessment scales—HAM-D, HAM-A, and YMRS— may reflect a pattern of clinical improvement from baseline (T0) to week four (T4). Notably, each scale showed a gradual decrease at every assessment point, indicating an improvement in symptoms over time, with effects that appeared to continue throughout the observation period. Consistent with these findings, recent evidence from a network meta-analysis of short-term trials in adults with bipolar depression indicates that lurasidone is associated with among the largest improvements in depressive symptoms, highlighting its relative efficacy compared with other second-generation antipsychotics (e.g., olanzapine, quetiapine), while some agents (e.g., cariprazine, aripiprazole, ziprasidone) showed more modest or negligible effects (38). These data support the potential value of lurasidone as a pharmacological option in this population, aligning our observational findings with broader clinical evidence.

When specifically evaluating the effectiveness of lurasidone on anxiety symptoms during the depressive episode, our results demonstrate a significant reduction in anxiety levels from baseline to the end of treatment, with a gradual and statistically significant improvement observed at each weekly assessment point. These effectiveness outcomes are comparable with those reported in previous studies in which a second-generation antipsychotic was introduced as an augmentation strategy in RBD, such as Teobaldi and colleagues’ study on cariprazine, which showed reductions in both depressive and anxious symptoms in real-world TRBD patients over the short-term period (30). However, when exploring whether patient profiles defined by baseline anxiety might be associated with a greater response to lurasidone, no significant differences were observed. In our sample, response to lurasidone appeared indifferent to the presence of moderate-to-high baseline anxiety levels, even when considering dose–response correlation. By contrast, in a study on cariprazine in patients with bipolar I depression, a *post hoc* analysis showed both significantly greater response with the 1.5 mg/d dose in patients with higher baseline anxiety and with 3 mg/d in patients with lower baseline anxiety, suggesting a dose-dependent effect of cariprazine on anxiety levels (39).

By the end of the observation period, one-third of patients in our sample (33.3%) achieved a clinical response, while remission was observed in only 3.3% of cases. These rates differ from those reported in some previous studies (25, 40), which may be attributable to differences in baseline clinical characteristics, such as the severity of depressive symptoms at study entry. As indicated by the initial HAM-D scores, patients in our cohort were experiencing moderate to severe depressive episodes, and their complexity—reflective of real-world clinical populations—likely reduced the probability of achieving full symptomatic remission within a four-week period. Our findings might indicate that lurasidone may have a greater effect on certain symptom domains of bipolar depression, while its impact on other aspects could be more limited, with effectiveness possibly linked to specific clinical features beyond symptom severity. This is also observed in the study by McIntyre and colleagues (38), which included individuals with bipolar depression characterized by mixed features that may have influenced both the therapeutic response and the overall outcomes observed.

The mean lurasidone dose observed in our sample (51.2 mg/day) may also help explain the low remission rates observed, as higher doses or longer observation periods at the same dose could potentially enhance therapeutic effects. Nevertheless, this dosing is in line with patterns commonly reported in routine clinical practice for non–treatment-resistant bipolar depression (41), suggesting that higher doses are not routinely utilized even in cases of TRBD. Supporting this observation, responders received significantly higher initial doses of lurasidone compared to non-responders; however, no significant difference was observed in dosing at week four. A higher baseline dose may have promoted more rapid activation of dopaminergic and serotonergic pathways, thereby enhancing the early antidepressant effects of lurasidone. It is also conceivable that clinicians, guided by clinical judgement, prescribed higher starting doses to patients perceived as more severely affected or better able to tolerate dose escalation, which may have indirectly influenced our outcomes. Nevertheless, the lack of a statistically significant difference in lurasidone dose at the end of the observation between groups suggests that therapeutic response within the administered dose range may not be strictly dose-dependent (41). Instead, individual variability in pharmacodynamic and pharmacokinetic parameters, alongside dose adjustments made during treatment in response to adverse effects or clinical improvement, may have mitigated any initial differences in dosing between groups.

In our sample, bipolar disorder subtype and baseline clinical scores did not significantly influence treatment response. Analyses comparing responders and non-responders are presented as exploratory. Clinical improvement was observed to be associated with lurasidone augmentation in combination with dual mood stabilizers and antipsychotics, suggesting a potentiated effect via complementary mechanisms (42). Notably, patients receiving antidepressants tended to show lower response rates, which may reflect more complex clinical presentations, refractory depressive episodes, or the presence of comorbidities (43, 44). Additionally, antidepressant use in BD may contribute to mood destabilization (45, 46), which could potentially reduce the effectiveness of adjunctive lurasidone. However, given the observational and uncontrolled nature of the study, no causal conclusions can be drawn from these associations.

In terms of tolerability, adverse events (AEs) were reported in 68.3% of the sample, with weight gain, tremor, and nausea emerging as the most frequently observed symptoms. Consistent with previous reports in clinical trials of lurasidone for bipolar depression (40), these AEs were generally considered related to lurasidone, although contributions from concomitant pharmacotherapy or their combination cannot be entirely excluded. The relatively high incidence of AEs in this naturalistic cohort may reflect the greater clinical heterogeneity and comorbidity burden typical of real-world populations, which are often under-represented in randomized controlled trials due to restrictive eligibility criteria.

These findings should be considered within the context of several methodological limitations. The retrospective observational design of the study and the relatively short duration of the study (four weeks) may limit the ability to infer causality or assess the durability of treatment effects. The absence of a control group further affects the internal validity and interpretability of the results. In this regard, the lack of a comparator arm also precludes any formal assessment of superiority over other antipsychotic treatments or the estimation of absolute risk reduction and number needed to treat. Additionally, the small sample size restricted the possibility of conducting stratified analyses based on specific clinical subgroups, and exploratory analyses comparing responders and non-responders may have been underpowered due to these smaller subgroup sizes. Moreover, the study did not allow for prespecified subgroup analyses according to prior treatments, metabolic characteristics, age-related cognitive decline, or suicidality, nor for formal correlation analyses across these domains. The inclusion of participants with a substantial burden of psychiatric comorbidities may have influenced both effectiveness and tolerability outcomes. Nonetheless, such comorbidities are highly prevalent among individuals with BD, thereby enhancing the ecological validity and applicability of the findings to real-world clinical populations. Similarly, the inclusion of background antidepressant treatments reflects routine clinical practice in complex and treatment-resistant bipolar depression, although it may have introduced additional variability such as rapid cycling. Furthermore, potential pharmacokinetic interactions—particularly those affecting lurasidone metabolism—were not systematically evaluated and may have contributed to interindividual variability in treatment response. Lastly, adverse events were recorded based exclusively on clinical documentation, without the implementation of standardized assessment tools, which may have limited the accuracy and consistency of tolerability reporting. These limitations highlight important opportunities for future prospective, controlled studies specifically designed to explore clinically relevant subgroups, including patients with metabolic vulnerability, cognitive impairment, or partial/non-response to prior treatments.

## 5 Conclusions

Despite the acknowledged limitations, this study provides novel evidence on the use of lurasidone in treatment-resistant bipolar depression (TRBD). Our findings already highlight some clinical profiles within this heterogeneous population for which lurasidone may represent an appropriate adjunctive treatment option. Nevertheless, its current off-label status in Europe and the modest remission rates observed reinforce the need for further research in larger and more diverse samples. Future investigations should aim to confirm these preliminary indications and to better define how lurasidone can be integrated into personalized treatment strategies, ultimately contributing to the development of precision approaches for patients who remain difficult to treat.

## Data Availability

The raw data supporting the conclusions of this article will be made available by the authors, without undue reservation.
